# Dr. Warren H. Loomis

**Published:** 1918-12

**Authors:** 


**Dr. Warren H. Loomis,** Long Island Medical College 1878, died at the City Hospital of Lockport Oct. 30, aged 63, of influenza. He was born in Palermo, N. Y. but had been in practice in Lockport since 1889. He attended the University of Buffalo before going to the college at which he graduated.

